# Prophylactic Closure of Mucosal Defect to Prevent Delayed Bleeding After Endoscopic Papillectomy

**DOI:** 10.1155/cjgh/5663582

**Published:** 2026-03-18

**Authors:** Jinpei Dong, Guigen Teng, Lu Zhang, Haixia Niu, Dapeng Bian, Qiushi Feng

**Affiliations:** ^1^ Department of Gastroenterology, Peking University First Hospital, Beijing, China, pkufh.com; ^2^ Department of Endoscopy, Peking University First Hospital, Beijing, China, pkufh.com; ^3^ Department of Hepatopancreatobiliary Surgery, Peking University First Hospital, Beijing, China, pkufh.com

## Abstract

**Background:**

Delayed bleeding is a serious complication of endoscopic papillectomy (EP). The aim of this study was to evaluate the efficacy of preventing delayed bleeding of clip after EP.

**Methods:**

Consecutive patients diagnosed with ampullary benign tumors who received EP from January 1^st^ 2013 to December 31^st^ 2024 were analyzed retrospectively. EP was performed with the snare polypectomy technique. Biliary duct stent and pancreatic duct stent were routinely placed. The resection site is closed completely by clips.

**Results:**

Fifty major papilla benign tumors patients with a mean age of 55.9 ± 10.7 years were found. All of the lesions were En bloc removed in a single endoscopic procedure. The pancreatic duct stents and biliary duct stents were successfully placed in 47 (94%) and 49 patients (98%), respectively. Two patients had positive horizontal margin, 35 patients had negative horizontal margin, and in 13 patients, horizontal margin was compromised by cautery effect. None of the patients had vertical positive margin, 39 patients had vertical negative margin, and in 11 patients, vertical margin was compromised by cautery effect. Post‐EP bleeding was observed in 2 patients (4%), which was classified as mild. Post‐EP hyperamylasemia was observed in 13 patients (26%), and post‐EP pancreatitis was observed in 7 patients (14%), 6 were mild pancreatitis and 1 was severe pancreatitis. Intraoperative perforation occurred in 1 patient, which was clamped with hemostatic clips. No cholangitis was observed. The mean follow‐up duration was 378.1 ± 305.7 days. Histologically confirmed recurrence at the resection site was detected in 6 patients (12%).

**Conclusions:**

This study demonstrated that preventive closure of the resection site by clips was technically feasible and effective in preventing delayed bleeding after EP, without increasing the risk of postprocedure pancreatitis and cholangitis.

## 1. Introduction

Ampullary neoplasm is a rare disease with an incidence of less than 1 per 100,000 per year and accounting for only 0.6%–0.8% of the digestive cancers, sex ratio of males to females was 1.5[1]. The symptoms are typically nonspecific or related to pancreatobiliary obstruction [1]. Owing to the accuracy of gastroduodenoscopy, the incidence of ampullary neoplasm seems to be increasing nowadays. Ampullary adenomas are likely to follow an adenoma‐to‐carcinoma sequence similar to colorectal adenocarcinoma, complete excision of the lesion is necessary. Pancreaticoduodenectomy and transduodenal papillectomy have long been the standard treatment for ampullary neoplasms based on the stage of the lesion, but the morbidity and mortality are relatively high [2]. Endoscopic papillectomy (EP) was first reported in 1983 as an endoscopic therapeutic modality for selected patients with ampullary neoplasms and had been established as a safe and minimally invasive alternative to pancreatoduodenectomy for the treatment of ampullary adenoma [3, 4]. However, EP is also associated with the risk of complications such as bleeding, pancreatitis, and perforation. Among them, delayed bleeding is a serious complication, ranged from 0% to 31.6% [5, 6]. Hemostatic clip was widely used after gastrointestinal polypectomy to reduce the risk of delayed bleeding; closure of the mucosal break may prevent delayed bleeding after EP. However, the evidence supporting the widespread application of clip to prevent delayed bleeding after EP is not sufficient. The aim of this study was to evaluate the efficacy of prevent delayed bleeding of clip after EP.

## 2. Methods

### 2.1. Patients

Consecutive patients diagnosed with ampullary benign tumors (adenoma or neuroendocrine tumor, G1) who received EP in our hospital from January 1^st^ 2013 to December 31^st^ 2024 were analyzed retrospectively. Only patients with completely closed wounds with clips after EP were enrolled. Patient demographics, clinical history, laboratory data, imaging data, and endoscopic pictures were collected.

#### 2.1.1. Indications for Endoscopic Treatment

(a) Biopsy pathology confirmed benign tumors (adenoma or neuroendocrine tumor, G1). (b) Absence of intraductal involvement. (c) No clinical features of invasive malignancy based on endoscopic assessment (e.g., ulceration or excessive friability).

#### 2.1.2. Preprocedural Evaluation

Endoscopic ultrasound (EUS), computed tomography (CT), and magnetic resonance imaging (MRI) were used to determine the above criteria. Colonoscopy was advised to perform in these patients to determine if colonic adenomatous polyps were coexisted. All of the patients discontinued anticoagulation or antiplatelet agents 7 days before EP.

#### 2.1.3. Endoscopic Procedures

**FIGURE 1:**
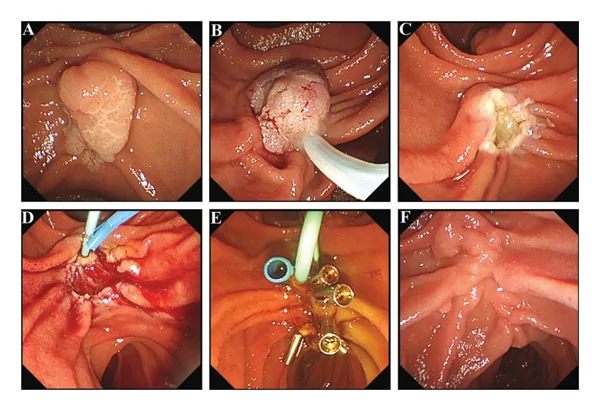
(A) Endoscopic view of the papillary lesion. (B, C) Endoscopic papillectomy was performed with snare polypectomy technique. (D) Biliary duct stent and pancreatic duct stent were placed. (E) The resection site was closed completely by clips. (F) Three months after papillectomy.

The duodenoscope type used to perform EP was TJF‐260V (Olympus Optical, Tokyo, Japan). Patients were sedated using intravenous propofol and remifentanil. EP was performed with snare polypectomy technique. The snare used was GPS‐21‐25‐180 (Endo‐Flex, Voerde, Germany). The electrosurgical unit used during the operation was the ESG‐400 (Olympus Optical, Tokyo, Japan) set to the PulseCut Fast mode (effect 3, output limit 40 W). Biliary duct stents and pancreatic duct stent were routinely placed through the major papilla resection site to reduce the risk of postprocedural cholangitis and pancreatitis. The major papilla resection site is closed completely by hemostatic clips (Sureclip, MICRO‐TECH (Nanjing, China) Co., Ltd) to reduce the risk of bleeding (Figure 1). The stents were typically removed within 1 month. Adverse events focused on delayed post‐EP bleeding, hyperamylasemia, pancreatitis, cholangitis, and perforation. Follow‐up endoscopy was scheduled after 1, 3, 6, and 12 months for the first year and then yearly for the following 5 years [3]. This study was approved by the institutional review board and the need for informed consent was waived (the ethic approval number: 2022521‐002).

### 2.2. Definition

Delayed post‐EP bleeding was defined as melena or hematemesis after EP, associated with a 3 g/dL decrease or more in hemoglobin levels [7]. The severity of the bleeding was graded as the following: (1) mild, clinical evidence of bleeding with a decrease in hemoglobin level, blood transfusion was not needed; (2) moderate, requiring endoscopic hemostasis and blood transfusion ( ≤ 4 units); and (3) severe, blood transfusion of 5 units or more were needed, or the need for radiological embolization or surgery for control of bleeding [8]. Cholangitis was defined as a combination of fever, abdominal pain, elevated liver function test, and/or serum bilirubin. Hyperamylasemia was defined as the elevation of serum amylase levels to more than 3 times the upper normal limit without abdominal pain and characteristic findings of acute pancreatitis on imaging. Pancreatitis was defined meet two of the following three features: (1) abdominal pain; (2) serum lipase activity (or amylase activity) at least 3 times greater than the upper limit of normal; and (3) characteristic findings of acute pancreatitis on CT or MRI or transabdominal ultrasonography [9]. Severity of pancreatitis was classified according to Revised Atlanta Classification [9]. Perforation was defined on the basis of symptoms and radiographic evidence of free intraperitoneal air. R0 margin was defined as microscopically negative, and R1 margin was defined as microscopically positive. Recurrence was defined as the presence of endoscopic and histological evidence of adenomatous tissue at the site of the resection [10].

## 3. Results

All of the patients were included after 2018. A total of 50 major papilla benign tumors patients (33 men and 17 women) with a mean age of 55.9 ± 10.7 years were found (Table 1). The major papilla benign tumors were found by gastroscopy in 39 patients, by CT or MRI in 9 patients, and by duodenoscopy in 2 patients. The major papilla benign tumors diameter was 13.6 ± 6.5 mm. Thirty‐four patients were performed colonoscopy and 25 of the patients combined with colon adenomas. All of the major papilla benign tumors were En bloc removed in a single endoscopic procedure. The pancreatic duct stents were successfully placed in 47 patients (47/50, 94%). The biliary duct stents were successfully placed in 49 patients (49/50, 98%). Two patients had positive horizontal margin, 35 patients had negative horizontal margin, and in 13 patients, horizontal margin was compromised by cautery effect. None of the patients had vertical positive margin, 39 patients had vertical negative margin, and in 11 patients, vertical margin was compromised by cautery effect. All of the patients were performed duodenoscopy before resection, histopathological examination of pre‐EP duodenoscopy biopsy of major papilla revealed low‐grade intraepithelial neoplasia (LGIN) in 45 patients (45/50, 86.9%), high‐grade intraepithelial neoplasia (HGIN) in 4 patients (4/50, 8%), and neuroendocrine tumors in 1 patient (1/50, 2%). Histopathological examination of endoscopic resection of major papilla revealed LGIN in 34 patients (34/50, 68%), HGIN in 15 patients (15/50, 30%), and neuroendocrine tumors in 1 patient (1/50, 2%) (Table 2).

**TABLE 1:** Baseline and procedural characteristics of the included patients.

Characteristics	Value
Sex	
Male	33
Female	17
Age (years)	55.9 ± 10.7
Major papilla benign tumors resection	
En bloc	50
Piecemeal	0
Lesion diameter (mm)	13.6 ± 6.5
Main pancreatic duct stent	
Success	47
Failure	3
Biliary duct stent	
Success	49
Failure	1
Horizontal margin	
R0	35
R1	2
Cauterization	13
Vertical margin	
R0	39
R1	0
Cauterization	11
Adverse events	
Pancreatitis	7
Hyperamylasemia	13
Bleeding	2
Cholangitis	0
Perforation	1

**TABLE 2:** Pathological characteristics of biopsy and resected lesions.

Pathology	Pre‐EP duodenoscopy biopsy	Post‐EP
LGIN	45	34
HGIN	4	15
Neuroendocrine tumors	1	1

*Note:* EP, endoscopic papillectomy; HGIN, high‐grade intraepithelial neoplasia; LGIN, low‐grade intraepithelial neoplasia.

All of the patients underwent EUS before EP, none of them with intraductal involvement. Thirty‐two patients underwent contrast‐enhanced CT before EP, and CT detected the major papilla lesions in 12 (12/32, 37.5%) patients. Thirteen patients underwent contrast‐enhanced MRI before EP, and MRI detected the major papilla lesions in 7 (7/13, 53.8%) patients. Post‐EP bleeding was observed in 2 patients (2/50, 4%), which was classified as mild. The bleeding was caused by an insecure hemostatic clip that detached prematurely. Both of them were successfully treated by hemostatic clipping again. Post‐EP hyperamylasemia was observed in 13 patients (13/50, 26%), and post‐EP pancreatitis was observed in 7 patients (7/50, 14%), 6 were mild pancreatitis and 1 was severe pancreatitis. All of the pancreatitis patients improved with conservative management. Intraoperative perforation occurred in 1 patient, the perforation wound was clamped with hemostatic clips. No cholangitis was observed. The mean follow‐up duration was 378.1 ± 305.7 days. Histologically confirmed recurrence at the resection site was detected in 6 patients (6/50, 12%).

## 4. Discussion

EP is a safe and minimally invasive therapy for the treatment of ampullary benign tumors (adenoma or neuroendocrine tumor, G1) [1, 3, 11]. The most common indication for EP is benign tumors without intraductal involvement in the pancreaticobiliary duct [6]. Most papilla benign tumors were found as an incidental finding in gastroscopy, but it is difficult to observe the whole lesion by gastroscopy due to the tangential angle involved. Side‐viewing duodenoscopy is superior to gastroscopy for optimal visualization of the papilla and evaluation of the feasibility of endoscopic resection [12]. Therefore, duodenoscopy was recommended to perform in patients suspected of papilla benign tumors.

The incidence of adverse events after EP was reported to be 6.1%–58.3%, including acute pancreatitis (0%–23.1%), delayed bleeding (0%–31.6%), perforation (0%–8.3%), and cholangitis (0%–7.3%) [5, 6]. Delayed bleeding after EP is a serious complication which is associated with the arterial supply of the major duodenal papilla. Papillary arteries originated from three sources: communicating arteries, the posterior pancreaticoduodenal arcade, and the anterior pancreaticoduodenal arcade [13]. The majority of papillary arteries were located in the 3 and 9 oʼclock positions, whereas only 10% and 11% of papillary arteries were located in the 10 and 11 oʼclock positions on microdissection and histology, respectively [13]. Prophylactic application of a hemostatic clip is associated with a significantly reduced rate of delayed bleeding after endoscopic sphincterotomy in high‐risk patients [14]. But another research found that delayed bleeding after EP occurs most usually from the frenulum area of the ampulla of Vater, and preventive closure of the frenulum area using a clip may have a protective effect on reducing delayed bleeding [15]. Actually, all of the sites of the mucosal break may have the risk of bleeding; only a clip place at the frenulum area cannot prevent delayed bleeding in all cases.

Intraoperative bleeding and lesion size ( ≥ 3 cm) were associated with increased risk of bleeding after EP, while endoscopic closure could reduce the risk [16]. Hemostatic clip was widely used after gastrointestinal polypectomy to reduce the risk of delayed bleeding [17], and a few studies evaluated the efficacy of clips to prevent bleeding after EP. A retrospective study found that prophylactic clipping of the mucosal break could reduce the risk of bleeding after EP, and a pancreatic stent was routinely placed but biliary stent was placed as an alternative [18]. Another retrospective study also found that clip was effective in preventing bleeding after EP, and a biliary stent was routinely placed but pancreatic duct stent was not placed [19]. A multicenter randomized trial evaluated the efficacy of newly designed clips to prevent bleeding after EP, and the incidence of delayed bleeding was numerically higher in the no‐clipping group than that in the clipping group (31.6% [95%CI 19.1–47.5] vs. 15.0% [95%CI 7.1–29.1]); however, the difference did not reach statistical significance (*p* = 0.14) [5]. Thus, the routinely use of pancreatic duct stent and biliary duct stent did not yield universal consensus, and the efficacy of clips to prevent bleeding after EP was not consistent across studies. In this study, the rate of delayed bleeding is lower compared with the previous studies [10, 20–22], which indicate that using clip close the resection site is an effective approach to prevent bleeding after EP.

Pancreatitis after EP are usually mild or moderate which can be treated conservatively, but severe pancreatitis cases and even death owing to severe pancreatitis after EP have also been reported [23, 24]. Prophylactic pancreatic duct stenting was proved to be effective in preventing pancreatitis and was, therefore, recommended by many guidelines and expert consensuses [2, 6, 25]. The incidence of pancreatitis after EP with clips close the iatrogenic ulcer range from 7.5% to 17.5%, with no significant differences with the no‐clip groups [5, 15, 18], which mainly benefit from prophylactic pancreatic duct stent placement. In this study, the rate of pancreatitis is 14%, consistent with the findings of previous literature reports without use clip, which indicate that clips did not increase the risk of postprocedure pancreatitis.

The effect of using biliary stent for preventing acute cholangitis remains controversial [6]. We support this approach for several reasons. First, preventing acute cholangitis. Second, decreasing the stimulation of bile to the resection surface, which may have a potential role for decreasing the risk of delayed perforation and delayed bleeding. Third, avoid closing the bile duct orifices when using clips to close the whole resection surface.

Traditional hemostatic clip was difficult to release by using duodenoscope; in recent years, some newly designed clips with an easy regrasping function and a controlled rotation orientation had largely overcome this difficulty [5, 26]. The main difficulty lies in avoiding to closing the pancreatic and bile duct orifices. Prophylactic pancreatic duct stent and bile duct stent placement could avoid the clips closing the pancreatic and bile duct orifices.

There are some limitations of this study. First, this is a retrospective, observational study and lack of control group. Second, the sample size of this study was relatively small and it was a single‐center experience. Finally, the follow‐up period was short in some of the patients; there is the potential to underestimate the recurrence rate. Therefore, multicenter randomized controlled prospective studies are required to further evaluate the efficacy of clips to prevent bleeding after EP.

In conclusion, our study demonstrated that preventive closure of the resection site by clips was technically feasible and effective in preventing delayed bleeding after EP, without increasing the risk of postprocedure pancreatitis and cholangitis [27].

## Author Contributions

Conception and design: Dapeng Bian and Qiushi Feng; acquisition of data: Jinpei Dong and Haixia Niu; analysis and interpretation of the data: Jinpei Dong, Lu Zhang, and Guigen Teng; drafting of the article: Jinpei Dong; critical revision of the article for important intellectual content: Dapeng Bian and Qiushi Feng; study supervision: Dapeng Bian and Qiushi Feng.

## Funding

No funding was received for this manuscript.

## Disclosure

All of the authors have read and approved the final version of the manuscript.

## Conflicts of Interest

The authors declare no conflicts of interest.

## Data Availability

Data sharing is not applicable to this article as no datasets were generated or analyzed during the current study.
